# Household and plot-level survey data on adoption, outcomes, and perceptions of early sown wheat and zero tillage in Northwest India

**DOI:** 10.1038/s41597-023-02401-x

**Published:** 2023-08-04

**Authors:** Dominik Naeher, Basma Albanna, Abhijeet Kumar, Sebastian Vollmer

**Affiliations:** 1 University of Goettingen, Department of Economics, Centre for Modern Indian Studies, Waldweg 26, 37073 Goettingen, Germany; 2 Ain Shams University, Faculty of Computer Science, Department of Information Systems, Cairo, Egypt

**Keywords:** Agriculture, Economics

## Abstract

This study collected evidence on the use of early sown wheat varieties and complementary zero tillage technologies in Northwest India. Detailed information on farmers’ knowledge, adoption decisions, personal experience, and perceptions of early sown wheat and zero tillage technologies were collected at the household level using different survey tools. Additional information on agricultural practices during the Rabi Season 2021/22 were collected at the plot level and geocoded. Overall, the dataset comprises responses from 1206 wheat farmers in 70 villages across 7 districts in Punjab and Haryana that were collected between September and October 2022. The villages were selected using stratified random sampling based on a sampling frame of 1722 communities that had been identified as predominantly wheat growing areas based on remote-sensing data from satellite images. The dataset provides rich information that may be used for assessing the diffusion and impact of recently developed wheat varieties designed for early sowing, identifying barriers to the wider adoption of these technologies, and informing policy making aimed at improving adoption and usage decisions of agricultural innovations.

## Background & Summary

A pressing problem in Northwest India are climate change-related increases in temperature and water shortages at the end of the wheat cropping season, which increasingly lead to widespread harvest failures and food insecurity among smallholder farmers in this region^1–3^. A potential solution to avoid crop damage during the terminal heat just before the wheat harvest season are new varieties that have higher early heat tolerance and thus can be sown early (in October and early November) compared to the conventional sowing (in late November and December)^4–6^. Early sowing then enables farmers to shift the cropping cycle (including harvest) forward, thereby reducing the risk of crop damage caused by terminal heat and water stress towards the end of the cropping season.

Such early sown wheat (ESW) varieties were recently developed and disseminated in different regions. For instance, following government-coordinated trials, three wheat varieties (DBW 187, DBW 303 and WH 1270) developed by the International Maize and Wheat Improvement Center (CIMMYT) were released in 2020 for October sowing under irrigated conditions of the North Western Plains Zone of India (to our knowledge, this was the first release of wheat varieties specifically for October sowing in India). However, the diffusion and impact of these ESW varieties remain largely unknown as systematic evidence on farmers’ adoption decisions and outcomes that would permit such an assessment is lacking. Our study was designed to start filling this gap and to identify potential barriers to the wider adoption of ESW varieties in India. Because there are important synergies between ESW and other agricultural technologies, such as zero tillage equipment^7,8^, we also collected information on the use and knowledge of these technologies.

Within the literature on agricultural technology adoption among smallholder farmers, a wide range of factors are considered as potential barriers to the adoption of modern farming technologies, including market imperfections related to finance and insurance^9,10^, limited access to complementary inputs (such as groundwater and affordable energy for irrigation)^11–14^, frictions in information and learning^15–17^, peer effects in social networks^18–21^, as well as other behavioural biases^22^. By collecting rich information both on actual adoption rates of ESW and respective agricultural outcomes, and on farmers’ knowledge and perceptions of ESW and zero tillage technologies (irrespective of whether they had personally used these technologies), our dataset may be useful for future research that investigates different channels and mechanism affecting ESW adoption, and ultimately to inform policy making aimed at improving adoption and usage decisions of these agricultural innovations.

There are a few other studies that have looked at early wheat sowing in India. Most of these studies focus on measuring the diffusion of early sowing based on sowing dates predicted from remote-sensing data^6,23–26^. Specifically, these dates are typically inferred from detectable changes in vegetation indices (green-up) obtained from satellite images combined with information from test farms on the average time wheat takes from sowing to green-up. While some of these studies also use remote-sensing data to provide estimates of the impact of early sowing on wheat yields, these data don’t typically allow to differentiate between the various wheat varieties used by farmers in this region, making it impossible to draw conclusions regarding the diffusion and impact of new ESW varieties. Moreover, we are not aware of any existing field data that combine direct (ground-verified) analysis of the relationship between ESW varieties, agricultural practices and outcomes, knowledge and attitudes related to ESW as well as characteristics of farms and farmers. By addressing this gap, our dataset can be used for assessing the diffusion and impact of specific wheat varieties (including those recently developed for early sowing) and to identify barriers to their wider adoption.

## Methods

Ethical approval for the study was received from the Ethics Committee of the University of Göttingen prior to the field work (date: 8 September 2022; approval number: none). Participants were provided with a written consent form and only interviewed if they stated their agreement to participate in the study and to the usage of the collected data in anonymized form for publication. The consent forms also specified that participation is voluntary and that, after choosing to participate, the participants have the right to opt out of the survey at any time or to not answer individual questions.

**Fig. 1:**
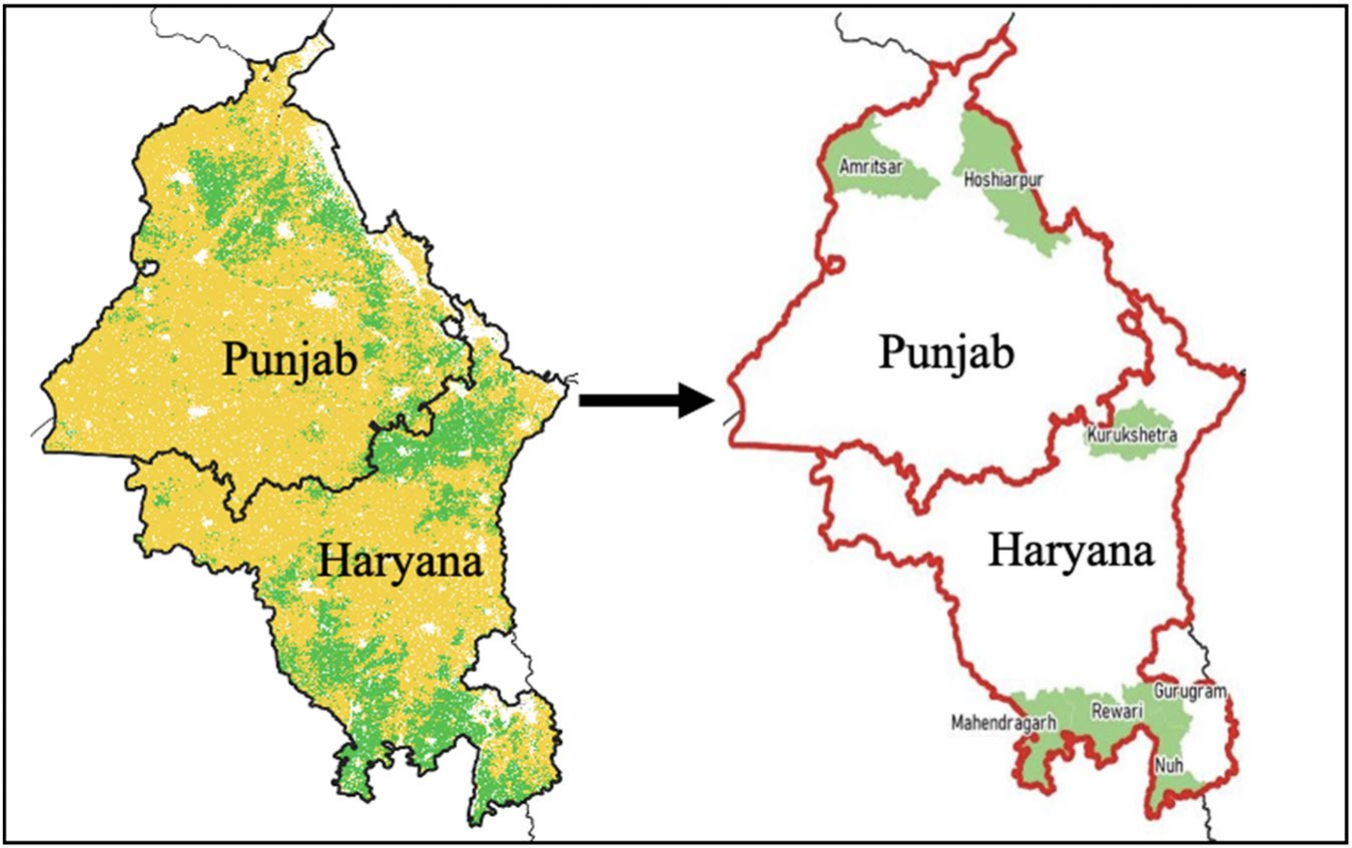
Study sampling frame. The map on the left shows (in green) communities exhibiting signs of early wheat sowing. These communities are concentrated in the districts selected for the study, as shown in the map on the right.

The sampling strategy relied on community boundary data (admin 5 units)^27^ as the primary sampling unit. The study population consisted of 9336 communities in Punjab and Haryana that had been identified as predominantly wheat growing areas through remote-sensing data. 1188 of these communities showed evidence of early wheat sowing, according to a remote-sensing data analysis technique outlined by Jain *et al*.^24^. These communities, hereafter referred to as ‘early sowing communities’, contained pixels of wheat area sown in October (and a prevalence from the period until mid-November). Whereas ‘regular sowing communities’ showed no signs of wheat pixels sown in October (and a prevalence from the period after mid-November). To reduce travel time during the field work and since the focus of this study is on early sowing wheat farmers and how they differ from the regular sowers, we decided to select districts that had a concentration of early sowing communities as shown in Fig. 1. Hence, in a first step seven districts were purposively selected to serve as the main sampling frame: two districts in Punjab (Amritsar, Hoshiarpur) and five districts in Haryana (Gurugram, Kurukshetra, Mahendragarh, Nuh, Rewari). These districts contained more than 70% (846) of the early sowing communities in the study population while also containing a sufficient number of regular sowing communities (n = 876).

In a second step, 70 communities were selected. The districts in Punjab contained 209 early sowing communities and 796 regular sowing communities, while the districts in Haryana contained 637 early sowing and 80 regular sowing communities. Our final sample comprised of 35 early sowing and 35 regular sowing communities that were selected based on stratified random sampling covering four main strata: Punjab early sowers, Punjab regular sowers, Haryana early sowers and Haryana regular sowers.

Finally, sampled communities were mapped to corresponding villages by matching the community name in the boundary dataset with the name of the village within its boundaries. For communities that comprised several villages, the most centrally located village was chosen for inclusion in the sample to increase the likelihood that the agricultural fields belonging to the village were located within the sampled community (as was to be expected, most fields we visited were located in the immediate surrounding of the villages where farmers lived). In the final step, the interviewer teams were instructed to select participants randomly from a list of wheat growing farmers provided by the village head (sarpanch). However, during the field work it turned out that many of the sample villages were so small that the interviewers managed to survey all wheat farmers in a village who were willing to participate in the study.

The survey instrument was organized in nine sections. In the first two sections, basic information about the respondent (Section A) and household characteristics (Section B) were recorded. In Sections C and D, interviewees were asked about their awareness of ESW and zero tillage technologies, perceptions about the impacts of using these technologies on different farm outcomes, sources of information, and previous adoption and usage experience. Importantly, these questions were asked to all farmers who reported to have heard of ESW or zero tillage solutions, irrespective of previous adoption decisions. In Sections E and F, detailed information on the cultivation practices on different agricultural plots were collected. The questions focused on the Rabi Season 2021/22 (the main season for wheat cultivation in India), though some question also referred to the previous season (Kharif 2021) and previous year (Rabi 2020/2021) to learn about farmers’ crop rotation practices. In Section E, general plot characteristics, such as location, area, and cultivated crops, were recorded. If the plot had been cultivated with wheat, additional information was collected on the type of wheat variety, sowing date (month and week), tillage method, harvested quantity, ownership status, plot management, and used farming inputs (including labour, irrigation, fertilizer, herbicide, pesticide), among others. This information was collected for the five largest plots cultivated in the Rabi Season 2021/22 (most of the surveyed farmers had one (52.2%) or two (28.4%) cultivated plots and only 3.7% reported to have five or more plots). Section F drilled down on one particular plot which was selected as the largest wheat plot with early sowing in the Rabi Season 2021/22 (or the largest wheat plot if the farmer had not used early wheat sowing in that season) based on the interviewees’ previous responses. For this plot, interviewees were asked to provide additional information regarding land preparation, residue use, seed type and source, harvest date, and allocation of labour to different agricultural tasks over the cropping cycle (including land preparation, sowing, weeding, irrigating, applying fertilizer and other inputs, harvesting, threshing, and grain cleaning). Section G collected information on respondents’ living standards, income, and asset ownership. In Section H, interviewees were asked about their perceptions towards different types of risks, including environmental and farming risks related to climate change. In the last section, respondents were asked to accompany the interviewer to the plot identified in Section F to record its GPS coordinates.

Each section used a range of different survey tools, including single-choice, multiple-choice, Likert-scale, and open-ended questions, as appropriate. The survey instrument was trialled before implementation and data was monitored for quality throughout the duration of the fieldwork (see the section on technical validation for more details).

Most farmers agreed to have the GPS coordinates of their plots recorded. Each interviewer team had a car with driver for their disposal in case a plot was too far for walking (though the average distance between households and plots in our sample was only 1.7 km). The interviewers had been trained in two ways of recording plot GPS coordinates. If it was possible to access the plot, then they would take the GPS coordinates at the centre of the plot. If walking on the plot was not feasible (e.g., due to flooding), then they would take the GPS coordinates at one edge of the plot and also record the compass direction towards the centre of the plot. Overall, the GPS coordinates of 515 plots were collected. Reasons for non-collection included inaccessibility (mostly due to flooded roads at the end of the monsoon season), time constraints, and distance to the plot (interviewers were instructed to skip plots located more than 10 km from the household).

To ensure anonymity of respondents, all variables that would allow for the identification of individuals or their villages have been removed from the dataset. In addition, the plot GPS coordinates reported in the dataset have been anonymized by first computing the village averages of the latitude and longitude of the plots belonging to each village and then adding random noise to the village average plot coordinates (with noise drawn from a uniform distribution between −0.1 and +0.1 arc degrees). While this helps to ensures anonymity of individual respondents, the manipulated coordinates still allow the dataset to be merged with other geocoded variables (such as rainfall or temperature estimates, for instance).

## Data Records

The dataset is accessible via the University of Göttingen data depository platform (Göttingen Research Online)^28^. The folder includes the dataset as a tab-file (which can be downloaded as a dta-file for use in Stata), the survey questionnaire in English (pdf-file), and a spreadsheet describing all variables and linking them to the respective survey item (xlsx-file). Variables in the dataset are labelled, referring mostly to the text of the respective survey item in the questionnaire. Binary variables corresponding to “Yes/No” questions were coded 1 (yes) and 2 (no). All other numerical variables corresponding to single- or multiple-choice questions carry value labels. Except for the above-mentioned manipulations made to ensure anonymity of the respondents, the data are in raw format.

## Technical Validation

In designing the questionnaire, the best practices currently applied in large household surveys such as the World Bank’s Living Standards Measurement Study - Integrated Surveys on Agriculture (LSMS-ISA)^29^ were observed. The survey instrument was trialled before implementation and data was monitored for quality on a daily basis throughout the duration of the fieldwork. Special attention was paid to adapting the questions, response items, and units of measurement to the local context. To ensure the accuracy of the survey instrument and its clarity to the respondents, the questionnaire was thoroughly tested, both before the start of the data collection with researchers at the University of Göttingen and agricultural experts in India, and in focus group discussions with local farmers during the pilot phase of the project. This resulted in several improvements in the way questions were formulated and adjustments in the response items for some questions.

To guard against the concern that the plot GPS coordinates collected during the last part of the survey might end up getting collected for a different plot than the one referred to in Section F, the survey tool was programmed so that interviewers had to confirm with the respondent, upon arrival at the plot, whether the plot had been planted with wheat in the Rabi Season 2021/22 and what the sowing date (month and week) had been. If the responses did not match the information recorded in Section F, then the interviewers pointed this out to the respondent and asked to be taken to the correct plot.

Another concern with the GPS coordinates may be precision. Of the 515 recorded plot coordinates, 70.8% had an accuracy that was better than 5 meters and 97.1% had an accuracy better than 10 meters (according to the GPS device), while only 9 cases (1.7%) had an accuracy worse than 15 meters.

## Usage Notes

There are no privacy or safety controls on public access to the data. Data for some variables are missing either due to questionnaire design or due to issues during data collection. Specifically, various questions were only asked conditional on the participants’ response(s) to other “trigger” questions (see the instructions in the questionnaire document). In all other cases, missing values are due either to the respondent’s unwillingness or inability to answer a particular question, or possibly because of data entry errors on the side of the enumerators (the data do not generally allow to distinguish between these reasons). No attempts were made to impute missing values following the completion of the data collection.

## Data Availability

No custom code was used in generating the dataset.
